# Efficacy of a Mouthwash Containing CHX and CPC in SARS-CoV-2–Positive Patients: A Randomized Controlled Clinical Trial

**DOI:** 10.1177/00220345231156415

**Published:** 2023-03-21

**Authors:** E.L. Bonn, A. Rohrhofer, F.X. Audebert, H. Lang, D.L. Auer, K.J. Scholz, P. Schuster, J.J. Wenzel, K.-A. Hiller, W. Buchalla, J.M. Gottsauner, V. Vielsmeier, B. Schmidt, F. Cieplik

**Affiliations:** 1 Department of Conservative Dentistry and Periodontology, University Hospital Regensburg, Regensburg, Germany; 2 Institute of Medical Microbiology and Hygiene, University of Regensburg, Regensburg, Germany; 3 Institute of Clinical Microbiology and Hygiene, University Hospital Regensburg, Regensburg, Germany; 4 Praxiszentrum Alte Mälzerei, Regensburg, Germany; 5 Department of Oral and Maxillofacial Surgery, University Hospital Regensburg, Regensburg, Germany; 6 Department of Otorhinolaryngology, University Hospital Regensburg, Regensburg, Germany

**Keywords:** COVID-19, antiseptic, cetylpyridinium chloride, chlorhexidine, mouthwash, SARS-CoV-2

## Abstract

Soon after the outbreak of the coronavirus disease 2019 (COVID-19) pandemic, preprocedural mouthwashes were recommended for temporarily reducing intraoral viral load and infectivity of individuals potentially infected with the severe acute respiratory syndrome coronavirus 2 (SARS-CoV-2) in order to protect medical personnel. Particularly, the antiseptic cetylpyridinium chloride (CPC) has shown virucidal effects against SARS-CoV-2 in vitro. Therefore, the aim of this randomized controlled clinical trial was to investigate the efficacy of a commercially available mouthwash containing CPC and chlorhexidine digluconate (CHX) at 0.05% each in SARS-CoV-2–positive patients as compared to a placebo mouthwash. Sixty-one patients who tested positive for SARS-CoV-2 with onset of symptoms within the last 72 h were included in this study. Oropharyngeal specimens were taken at baseline, whereupon patients had to gargle mouth and throat with 20 mL test or placebo (0.9% NaCl) mouthwash for 60 s. After 30 min, further oropharyngeal specimens were collected. Viral load was analyzed by quantitative reverse transcriptase polymerase chain reaction, and infectivity of oropharyngeal specimens was analyzed by virus rescue in cell culture and quantified via determination of tissue culture infectious doses 50% (TCID_50_). Data were analyzed nonparametrically (α = 0.05). Viral load slightly but significantly decreased upon gargling in the test group (*P* = 0.0435) but not in the placebo group. Viral infectivity as measured by TCID_50_ also significantly decreased in the test group (*P* = 0.0313), whereas there was no significant effect but a trend in the placebo group. Furthermore, it was found that the specimens from patients with a vaccine booster exhibited significantly lower infectivity at baseline as compared to those without vaccine booster (*P* = 0.0231). This study indicates that a preprocedural mouthwash containing CPC and CHX could slightly but significantly reduce the viral load and infectivity in SARS-CoV-2–positive patients. Further studies are needed to corroborate these results and investigate whether the observed reductions in viral load and infectivity could translate into clinically useful effects in reducing COVID-19 transmission (German Clinical Trials Register DRKS00027812).

## Introduction

Coronavirus disease 2019 (COVID-19) is caused by severe acute respiratory syndrome coronavirus 2 (SARS-CoV-2), an enveloped single-stranded RNA virus, which is primarily transmitted by direct contact or airborne transmission via droplets and aerosols (van Doremalen et al. 2020). It is known that the viral load of SARS-CoV-2 is exceptionally high in the oral cavity and the pharynx due to high abundance of cells expressing the transmembrane angiotensin-converting enzyme 2 (ACE-2), which is necessary for entrance and fusion of the SARS-CoV-2 viral envelope with target cells (Herrera et al. 2020; Peng et al. 2020; Wölfel et al. 2020; Zou et al. 2020).

Therefore, right after onset of the COVID-19 pandemic, it was discussed that health care professionals (HCPs) from disciplines with close patient contact, where no face masks can be worn by the patients during examination and therapy, could be at high risk for nosocomial infection with SARS-CoV-2, including dentists, maxillofacial surgeons, and otorhinolaryngologists (Meng et al. 2020; Peng et al. 2020). Although recent data suggest that dental practice personnel are at no higher risk for infection with SARS-CoV-2 as compared to the general public (Araujo et al. 2021; Mksoud et al. 2022), there are also reports stating high infection risks for dentists as well as for HCPs from other specialties (Nguyen et al. 2020; Sarapultseva et al. 2021). Among other measures such as personal protective equipment, preprocedural mouthwashes have been discussed and recommended from the early stages of the pandemic for temporarily reducing the intraoral viral load and infectivity in SARS-CoV-2–positive individuals (Gottsauner et al. 2020; Herrera et al. 2020; Meister et al. 2020; Meng et al. 2020; Peng et al. 2020; Meister et al. 2022). While some studies could quickly demonstrate that several antiseptics had a high virucidal efficacy to SARS-CoV-2 in vitro (Bidra et al. 2020; Meister et al. 2020; Muñoz-Basagoiti et al. 2021; Meister et al. 2022), the clinical translation of these in vitro results is still not clear. The few clinical trials that have been conducted come to different conclusions: some advocate the use of antiseptic mouthwashes in SARS-CoV-2–positive individuals, while others do not (Gottsauner et al. 2020; Chaudhary et al. 2021; Domênico et al. 2021; Ferrer et al. 2021; Huang and Huang 2021; Seneviratne et al. 2021; Alemany et al. 2022; Barrueco et al. 2022; Meister et al. 2022). This is mainly due to the lack of virus rescue in cell culture, which is essential to assess viral infectivity, whereas quantitative reverse transcriptase polymerase chain reaction (qRT-PCR) just detects viral RNA copies by their presence but cannot give an indication on whether these particles are infectious or not (Ferrer et al. 2021; Barrueco et al. 2022; Cieplik and Jakubovics 2022; Meister et al. 2022).

Chlorhexidine digluconate (CHX) and cetylpyridinium chloride (CPC) can be considered the antiseptics that are most commonly used in dental practice (Cieplik et al. 2019; Marui et al. 2019; Mao et al. 2020). Besides a high antibacterial efficacy against planktonic bacteria in saliva and aerosols (Marui et al. 2019), particularly CPC yielded promising in vitro data against SARS-CoV-2, based on disruption of the viral envelope, which prevents fusion with the target cell (Koch-Heier et al. 2021; Muñoz-Basagoiti et al. 2021; Meister et al. 2022).

Therefore, the aim of the present study was to investigate the efficacy of a commercially available mouthwash containing CHX and CPC regarding the reduction of the intraoral viral load and infectivity in SARS-CoV-2–positive patients as compared to a placebo mouthwash by using qRT-PCR for detection of viral load and virus rescue in cell culture for evaluation of viral infectivity. In addition, viral load and infectivity were also assessed with respect to the COVID-19 vaccination status of the patients.

## Materials and Methods

### Study Design and Ethical Considerations

The present study is a prospective randomized controlled clinical trial investigating the efficacy of a commercially available mouthwash containing 0.05% CPC and 0.05% CHX (PerioAid Active Control; Dentaid SL) as compared to a placebo mouthwash (0.9% NaCl) on reducing the intraoral viral load and infectivity in SARS-CoV-2–positive patients.

The study design followed the requirements outlined in the Consolidated Standards of Reporting Trials (CONSORT) 2010 statement and was approved by the internal review board of the University of Regensburg (ref. 20-1787_3-101) in accordance with the 1964 Declaration of Helsinki and its later amendments or comparable ethical standards. The study has been prospectively registered at the German Clinical Trials Register (ref. DRKS00027812).

### Inclusion and Exclusion Criteria

Patients admitted to a private practice with a special focus on infectious diseases (Praxiszentrum Alte Mälzerei, Regensburg, Germany) were screened for inclusion in this study. To be included, patients had to exhibit COVID-19–like symptoms for not longer than 72 h as well as a positive antigen point-of-care test (SARS-CoV-2 Rapid Antigen Test; Roche) at the time of inclusion in the study. Exclusion criteria were indication for intubation or mechanical ventilation and severe stomatitis. Written informed consent was obtained from all individual participants included in the study. Besides demographic data such as age and gender, also anamnestic data such as COVID-19 vaccination status, history of infection with SARS-CoV-2, and time periods since the last vaccine shot or infection were recorded.

### Clinical Procedures

Patients were randomly assigned to test or placebo group using a randomization table generated by SPSS, version 26 (SPSS, Inc.), immediately after the SARS-CoV-2 antigen test turned out to be positive. Then, baseline (BL) oropharyngeal specimens were acquired by letting the patients gargle their mouth and throat with 10 mL 0.9% NaCl for 20 s. These specimens were used for qRT-PCR–based confirmation of infection with SARS-CoV-2 and genotyping and as baseline specimens for determination of viral load and determination of tissue culture infection doses 50% (TCID_50_). Immediately afterward, patients had to rinse their mouth and throat with 20 mL test (PerioAid Active Control; Dentaid SL) or placebo mouthwash (0.9% NaCl) by gargling their mouth and their throat for 60 s, whereby they were blinded to the respective group. Thirty minutes after gargling, a further oropharyngeal specimen was obtained by asking the patients to gargle their mouth and throat with 10 mL 0.9% NaCl for 20 s. The 30-min postrinse period was chosen to reflect routine dental and otorhinolaryngological procedures. The investigators of qRT-PCR, genotyping, and virus culture experiments were blinded to the respective group.

### QRT-PCR–Based Analysis of Viral Load and Genotyping

Nucleic acids were isolated from oropharyngeal specimens using EZ1 Virus Mini Kit v2.0 with EZ1 Advanced XL system (Qiagen), as described previously (Gottsauner et al. 2020; Meister et al. 2022). Viral RNA was amplified using a published SARS-CoV-2 E gene qRT-PCR protocol (Corman et al. 2020) on the StepOnePlus qRT-PCR System (Thermo Fisher Scientific). For quantification, a standard curve was prepared from in vitro transcribed RNA. Genotyping of SARS-CoV-2 RNA was performed using specific VirSNiP assays (TIB Molbiol). These kits allow identifying the virus genotype by testing for characteristic single-nucleotide polymorphisms in the spike gene by means of qRT-PCR and melting curve analysis with specific fluorescent molecular probes. Spike positions p.E484A (g.23013A > C) p.L452R (g.22917T > G) and p.S371L/p.S373P (g.22673T > C, g.22674C > T) were analyzed.

### Virus Culture and Determination of TCID_50_

Virus culture and determination of TCID_50_ was performed as described before (Meister et al. 2022). SARS-CoV-2 was isolated from all oropharyngeal specimens at BL and 30 min after gargling. Vero cells were cultivated in Dulbecco’s modified Eagle’s medium (DMEM) supplemented with 10% heat-inactivated fetal calf serum (Sigma-Aldrich), 90 U/mL streptomycin, 0.3 mg/mL glutamine, 200 U/mL penicillin, and 2.5 μg/mL amphotericin B (PAN Biotech). Viral titers of the oropharyngeal specimens were determined by endpoint dilution on Vero cells and calculating TCID_50_ as plaque-forming units (PFU) per mL.

### Data Analysis

Data are reported as median values (with first and third quartiles) or proportions (numbers of patients), respectively. Data were analyzed statistically by applying nonparametric procedures using GraphPad Prism, version 9 (GraphPad Software). Mann–Whitney *U* tests or χ^2^ tests were used for pairwise comparisons between independent groups, while Wilcoxon signed-rank tests were used for pairwise comparisons for related groups over time between BL and 30 min. The significance level was set at α = 0.05.

## Results

### Patient Population

**Figure 1.**
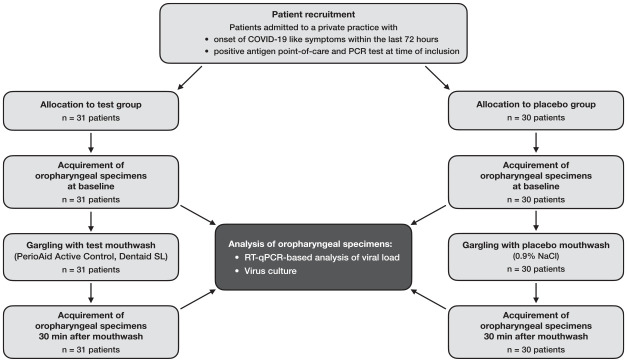
Flow of patients through the stages of this study.

Between January 4, 2022, and February 22, 2022, 61 SARS-CoV-2–positive patients were included in this study, of whom 31 were randomly assigned to the test group and 30 to the placebo group. Figure 1 shows the CONSORT flow of patients for this study, and the Table summarizes the patient characteristics of all individual patients included in this study. The median (first, third quartile) age was 29 (25, 42) years for all included patients. Of the participants, 86.9% had received at least 1 COVID-19 vaccination dose, but most had received 2 or 3 vaccine shots. Seven patients had a history of a SARS-CoV-2 infection (confirmed by qRT-PCR), resulting in a significant difference between test and placebo groups (*P* = 0.04). Besides that, there were no significant differences between test and placebo groups regarding patient characteristics.

**Table.** Patient Characteristics.

Characteristic	All Patients (*n* = 61)	Test Group (*n* = 31)	Placebo Group (*n* = 30)	Significant Differences
Age, median (first, third quartile), y	29 (25, 42)	29 (26, 36.5)	29.5 (25, 48)	—
Sex				
Female	49.2 (30)	45.2 (14)	53.3 (16)	—
Male	50.8 (31)	54.8 (17)	46.7 (14)	
Vaccination status				
Vaccinated	86.9 (53)	93.5 (29)	80 (24)	—
3 vaccine shots	57.4 (35)	67.7 (21)	46.7 (14)	
2 vaccine shots	26.2 (16)	25.8 (8)	26.7 (8)	
1 vaccine shot	3.3 (2)	—	6.7 (2)	
Nonvaccinated	13.1 (8)	6.5 (2)	20 (6)	
Period since last vaccination, median (first, third quartile), d	67 (46, 101)	62 (43, 87)	73.5 (49, 122.3)	—
COVID-19 infection status				
Previous infection	11.5 (7)	3.2 (1)	20 (6)	0.04
No previous infection	88.5 (54)	96.8 (30)	80 (24)	
Period since previous COVID-19 infection, median (first, third quartile), d	118 (99, 228.5)	392^a^	117.5 (90, 148.8)	NA

Values are presented as % (*n*) unless otherwise indicated. Statistically significant differences from pairwise comparisons between test and placebo group (χ^2^ tests; α = 0.05) are indicated. *P* value, significant (*P* ≤ 0.05).

COVID-19, coronavirus disease 2019; NA, not applicable; —, not significant (*P* > 0.05).

a Single values (for *n* = 1).

### Viral Load

**Figure 2.**
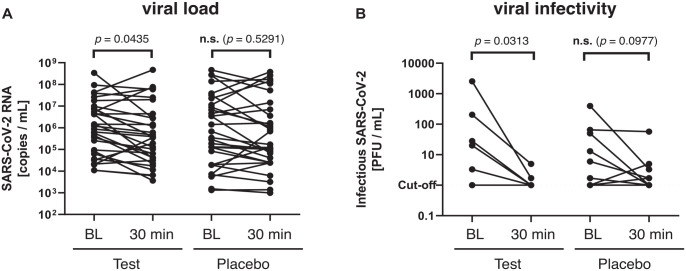
Effects of test and placebo mouthwash on viral load and infectivity. (**A**) Severe acute respiratory syndrome coronavirus 2 (SARS-CoV-2) RNA (copies per mL) as detected by quantitative reverse transcriptase polymerase chain reaction (qRT-PCR) from the oropharyngeal specimens taken at baseline (BL) and 30 min after the test (*n* = 31) or placebo mouthwash (*n* = 30). There was a statistically significant reduction in viral load in the test group between BL and 30 min (Wilcoxon signed-rank test; α = 0.05). (**B**) Infectious SARS-CoV-2 particles (plaque-forming units [PFU] per mL) as determined by virus culture from the oropharyngeal specimens taken at BL and 30 min after the test (*n* = 6) or placebo mouthwash (*n* = 9). There was a statistically significant reduction in viral infectivity in the test group and a trend in the placebo group (Wilcoxon signed-rank test; α = 0.05).

The assessment of the viral load via qRT-PCR showed a median (first, third quartile) viral load of 1.2 × 10^6^ (8.3 × 10^4^; 7.5 × 10^6^) viral RNA copies/mL for the test group and of 5.1 × 10^5^ (2 × 10^4^; 1.4 × 10^7^) copies/mL for the placebo group at baseline. The specimens after 30 min exhibited a median viral load of 3.7 × 10^5^ (3.8 × 10^4^; 2.8 × 10^6^) copies/mL for the test group and 1.5 × 10^5^ (2.5 × 10^4^; 8.9 × 10^6^) copies/mL for the placebo group. There were no significant differences between groups at either time point. However, as compared to baseline, there was a decrease by 0.5 log_10_ in both groups after the mouthwash, which was found significant in the test group (*P* = 0.0435) but not in the placebo group (*P* = 0.5291; Fig. 2A).

### Viral Genotyping

In 10 of the 61 patients, viral genotyping was performed by testing for characteristic single-nucleotide polymorphisms in the SARS-CoV-2 spike gene. The samples of these patients harbored mutations specific for variant-of-concern (VOC) omicron (E484A and/or S371L/P in combination with wildtype at position L452), being representative for the ongoing omicron wave in Germany at the time of patient recruitment.

### Viral Infectivity

The viral infectivity was assessed by viral culture and determination of TCID_50_. At baseline, 6 specimens of the test group and 9 of the placebo group showed replicating virus. Accordingly, a median (first, third quartile) baseline TCID_50_ of 24 (7.5, 160.8) PFU/mL was detected for the test group and 6 (1, 50) PFU/mL for the placebo group. The specimens 30 min after the mouthwash showed a median TCID_50_ of 1 (1, 1.5) PFU/mL for the test group and 1.7 (1, 3.3) PFU/mL for the placebo group. There were no significant differences between groups at either time point. However, as compared to baseline, there was a significant decrease in viral infectivity in the test group by 1.4 log_10_ (*P* = 0.0313), whereas there was a nonsignificant decrease by 0.6 log_10_ in the placebo group (*P* = 0.0977) (Fig. 2B).

### Effects of Vaccination Status on Baseline Viral Load and Infectivity

**Figure 3.**
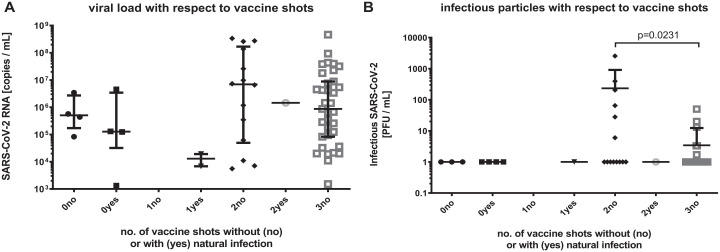
Viral load and infectivity with respect to vaccination status. (**A**) Severe acute respiratory syndrome coronavirus 2 (SARS-CoV-2) RNA (copies per mL) as detected by quantitative reverse transcriptase polymerase chain reaction (qRT-PCR) from the baseline oropharyngeal specimens. Depiction of numbers of vaccine shots (0, 1, 2, 3) and history of natural infection (yes, no). (**B**) Infectious SARS-CoV-2 particles (plaque-forming units [PFU] per mL) as determined by virus culture from the baseline oropharyngeal specimens. Depiction of numbers of vaccine shots (0, 1, 2, 3) and history of natural infection (yes, no). Oropharyngeal specimens taken from individuals with 3 vaccine shots exhibited significantly less infectious particles than those with just 2 vaccine shots (Mann–Whitney *U* test; α = 0.05).

When comparing the baseline viral load and infectivity of patients with 2 vaccine shots to those with 3 vaccine shots, the latter exhibited a slightly but not significantly lower viral load but a significantly decreased viral infectivity (*P* = 0.0231; Fig. 3).

## Discussion

Although the use of mouthwashes has been discussed as a potential measure to reduce transmission of SARS-CoV-2 since the beginning of the COVID-19 pandemic, the clinical evidence is still limited, particularly as there are only very few studies assessing their effects on viral infectivity by virus rescue in cell culture (Gottsauner et al. 2020; Alemany et al. 2022; Barrueco et al. 2022; Meister et al. 2022). Thus, we conducted a randomized placebo-controlled clinical trial investigating a commercially available mouthwash containing 0.05% CPC and 0.05% CHX in SARS-CoV-2–positive patients and analyzed not only the viral load by qRT-PCR but also the viral infectivity by virus rescue in cell culture and determination of TCID_50_.

CHX and CPC can be considered gold-standard antiseptics in dentistry and have a broad range of application in mouthwashes or other oral care products (Cieplik et al. 2019; Mao et al. 2020). While it is well known that the antibacterial efficacy of cationic antiseptics is limited toward mature oral biofilms mostly due to the biofilm matrix, they are highly effective toward planktonic bacteria such as those in saliva (Cieplik et al. 2019; Mao et al. 2020; Jakubovics et al. 2021). Accordingly, it has been shown that preprocedural mouthwashes with CHX or CPC can reduce the numbers of viable bacteria in aerosols generated during dental treatment, thus potentially contributing to infection control in dental practice (Marui et al. 2019; Koletsi et al. 2020).

According to available results from in vitro studies, CPC has shown promising antiviral effects against SARS-CoV-2 (Koch-Heier et al. 2021; Muñoz-Basagoiti et al. 2021; Meister et al. 2022). For instance, Meister et al. evaluated a wide range of antiseptics for their efficacy to SARS-CoV-2 in vitro and found that CPC, benzalkonium chloride (BAC), polyvenylpyrrolidone iodine (PVP-I), and a mixture of surfactants showed strong dose-dependent reductions of SARS-CoV-2 infectivity, exerted through disruption of the virus envelope (Meister et al. 2022). BAC was then investigated as mouthwash in a randomized placebo-controlled clinical trial, but the high antiviral efficacy found in vitro translated to only mild and nonsignificant effects on viral load and infectivity in clinics, probably related to the rather low sample size, particularly for virus rescue in cell culture (Meister et al. 2022). However, it was proposed that combinations of antiseptics as usually sold in commercial products may yield higher efficacy (Meister et al. 2022) and could provide synergistic effects (Koch-Heier et al. 2021).

When assessing the effects of the mouthwash on viral loads, we found slight reductions in both groups. Despite statistical significance found for the 0.8 log_10_ reduction in the test group, this may mainly be attributed to the mechanical effect inherent to rinsing and gargling rather than to antiseptic action, as we observed a similar trend (reduction by 0.4 log_10_) in the placebo group gargling with 0.9% NaCl. Furthermore, the baseline viral load in the test group was about 0.5 log_10_ RNA copies/mL higher than in the placebo group, which may have also influenced the results and could be considered a potential limitation of the randomization process based on a computer-generated randomization table that did not consider the vaccination status and history of COVID-19 infection in the included patients. Barrueco et al. (2022) also detected a significant decrease in the mean values of viral load 1 h after rinsing only in the placebo group. Furthermore, CPC does not affect the integrity of viral RNA but the viral envelope (Muñoz-Basagoiti et al. 2021; Meister et al. 2022). Therefore, qRT-PCR cannot be considered a sufficient method to evaluate the clinical efficacy of antiseptics such as CPC against SARS-CoV-2 (Gottsauner et al. 2020; Ferrer et al. 2021; Barrueco et al. 2022; Cieplik and Jakubovics 2022; Meister et al. 2022).

The successful rescue of SARS-CoV-2 in cell culture is very challenging and strongly correlates with high viral load in the samples (>10^7^ RNA copies/mL), positive detection of viral antigen, and short period after symptom onset (Gniazdowski et al. 2020; Wölfel et al. 2020; Buder et al. 2021; Hakki et al. 2022). Therefore, positive antigen point-of-care tests and symptom onset within the last 72 h were inclusion criteria for the present study, but still SARS-CoV-2 could not be successfully cultured from all baseline oropharyngeal specimens, reducing sample size to 6 (test group) or 9 patients (control group), which can be considered a limitation of the present study. When comparing viral infectivity at baseline and 30 min after the test mouthwash, there was a significant decrease by 1.4 log_10_. Very recently, Barrueco et al. (2022) assessed the effects of 4 commercially available antiseptic mouthwashes, including 1 containing 0.07% CPC in a randomized controlled clinical trial, and performed virus rescue in cell culture for assessing viral infectivity from saliva specimens obtained at baseline and 30 or 60 min following the mouthwash. They observed a significant decrease of 1.5 log genome copies/mL 60 min after the CPC-containing mouthwash, similar to our results 30 min after the mouthwash (1.4 log_10_ PFU/mL), but found no reduction at the shorter period of 30 min (Barrueco et al. 2022). These results are in line with another recent study by Alemany et al. (2022), who performed a randomized controlled clinical trial investigating a commercially available mouthwash containing 0.07% CPC as active ingredient. Despite not performing virus culture, they described a surrogate for virus particle degradation by modifying a commercially available enzyme-linked immunosorbent assay for the SARS-CoV-2 nucleocapsid protein. By omitting the step of membrane lysis, increased detection of nucleocapsid indicates destruction of the viral envelope by the mouthwash or its active ingredient CPC. Indeed, the levels of SARS-CoV-2 nucleocapsid protein were significantly higher in the test group 1 and 3 h following the mouthwash than in the placebo group (Alemany et al. 2022). In synopsis of these 2 studies and the present one, there is growing evidence that preprocedural mouthwashes containing CPC may exert some antiviral effects on SARS-CoV-2. Notably, we observed at least a trend toward reduced infectivity after gargling with 0.9% NaCl, so the process of gargling alone seems to have an effect. It must further be considered that it is still unclear whether the observed reductions of SARS-CoV-2 infectivity after gargling with mouthwashes containing CPC or other active ingredients can lead to clinically relevant reductions in the risk of transmission of SARS-CoV-2 (Cieplik and Jakubovics 2022). Furthermore, it must be kept in mind that the frequent use of antiseptics may also exert some negative effects such as inducing potentially detrimental ecological shifts in the oral microbiota (Mao et al. 2022) or development of antiseptic resistance in oral bacteria (Verspecht et al. 2019; Auer et al. 2022; Muehler et al. 2022).

Besides the main scope of the present study, we also assessed the viral load and viral infectivity of the baseline oropharyngeal specimens with respect to the COVID-19 vaccination status of the included patients. Interestingly, we found that the samples from individuals with 3 vaccine shots exhibited significantly less infectious particles than those with just 2 vaccine shots. IgA and IgG neutralizing antibodies (nAbs) have been detected in the saliva of patients vaccinated with 2 shots of messenger RNA–based vaccines (Ketas et al. 2021; Mostaghimi et al. 2021). However, while the nAb titers continuously decrease over a period of 6 to 8 mo following the second vaccination dose, a third vaccine shot (as “booster” about 6 mo after the second shot) increases the nAb titers again for a certain period (Sette and Crotty 2022). Since the interval to the last vaccination was shorter in patients with 3 shots than in those with 2, this could explain the lower infectivity found in samples from those patients who had received this booster vaccination.

## Conclusion

The present study indicates that gargling mouth and throat with a commercial mouthwash containing 0.05% CPC and 0.05% CHX could slightly but significantly reduce viral load in SARS-CoV-2–positive patients. Despite a small sample size, the test mouthwash also significantly reduced viral infectivity, while notably, gargling with 0.9% NaCl also had a slight effect. These findings add some further evidence for a potential effect of CPC-containing mouthwashes on reducing SARS-CoV-2 infectivity, although further studies with larger sample sizes are needed to corroborate these results.

## Author Contributions

E.L. Bonn, contributed to data acquisition, analysis and interpretation, drafted and critically revised the manuscript; A. Rohrhofer, contributed to data analysis, critically revised the manuscript; F.-X. Audebert, contributed to conception and design, data acquisition and interpretation, critically revised the manuscript; H. Lang, contributed to conception and design, data acquisition, critically revised the manuscript; D.L. Auer, contributed to acquisition, analysis and interpretation, critically revised the manuscript; K.J. Scholz, P. Schuster, B. Schmidt, contributed to conception and design, data analysis and interpretation, critically revised the manuscript; J.J. Wenzel, K.-A. Hiller, contributed to data analysis and interpretation, critically revised the manuscript; W. Buchalla, J.-M. Gottsauner, V. Vielsmeier, contributed to conception and design, data interpretation, critically revised the manuscript; F. Cieplik, contributed to conception and design, data analysis and interpretation, drafted and critically revised the manuscript. All authors gave their final approval and agree to be accountable for all aspects of the work.
